# Network Analysis Identifies Gene Regulatory Network Indicating the Role of RUNX1 in Human Intervertebral Disc Degeneration

**DOI:** 10.3390/genes11070771

**Published:** 2020-07-09

**Authors:** Nazir M. Khan, Martha E Diaz-Hernandez, Steven M. Presciutti, Hicham Drissi

**Affiliations:** 1 Department of Orthopaedics, Emory University, Atlanta, GA 30322, USA; martha.elena.diaz.hernandez@emory.edu (M.E.D.-H.); steven.presciutti@emory.edu (S.M.P.); hicham.drissi@emory.edu (H.D.); 2 Atlanta VA Medical Center, Decatur, GA 30033, USA

**Keywords:** interaction network, pathway enrichment, regulatory-network, gene-ontology, transcription factor, network cluster, RUNX1

## Abstract

Intervertebral disc (IVD) degeneration (IDD) is a multifactorial physiological process which is often associated with lower back pain. Previous studies have identified some molecular markers associated with disc degeneration, which despite their significant contributions, have provided limited insight into the etiology of IDD. In this study, we utilized a network medicine approach to uncover potential molecular mediators of IDD. Our systematic analyses of IDD associated with 284 genes included functional annotation clustering, interaction networks, network cluster analysis and Transcription factors (TFs)-target gene network analysis. The functional enrichment and protein–protein interaction network analysis highlighted the role of inflammatory genes and cytokine/chemokine signaling in IDD. Moreover, sub-network analysis identified significant clusters possessing organized networks of 24 cytokine and chemokine genes, which may be considered as key modulators for IDD. The expression of these genes was validated in independent microarray datasets. In addition, the regulatory network analysis identified the role of multiple transcription factors, with RUNX1 being a master regulator in the pathogenesis of IDD. Our analyses highlighted the role of cytokine genes and interacting pathways in IDD and further improved our understanding of the genetic mechanisms underlying IDD.

## 1. Introduction

Intervertebral disc (IVD) degeneration (IDD) is a multifactorial physiological process, which is associated with low back pain, a major public health problem affecting millions of people globally and now considered the leading cause of disability worldwide [1,2,3,4]. The etiology of IDD is complex, and is mainly characterized by an imbalance between synthesis and degradation of extracellular matrices [5,6]. The major contributing factors affecting the pathophysiology of IDD include genetic predisposition, aging, smoking and alcohol consumption and unhealthy lifestyle conditions including obesity and diabetes [7,8]. The current knowledge of the molecular basis of pathophysiological processes involved in the initiation and progression of IDD remains elusive. Therefore, better understanding of the molecular cascade underlying disc degeneration will help designing successful therapeutic strategies.

Recent studies using classic experimental and array-based approaches have identified some of the genes and molecular factors involved in the pathophysiology of IDD [6,9,10]. However, these studies only provided preliminary analyses to link these changes in gene expression with IDD pathogenesis. Additionally, these genes do not play an independent role in the genome, instead they work in synergy with other genes to form pathway networks based on their intricate interactions and execute biological functions [11,12]. Therefore, it is speculated that a large number of genes and their interaction with each other are involved in the pathogenesis of IDD. We therefore postulated that an integrated analysis of these candidate genes would provide a holistic view of their genetic contribution to IDD onset and progression.

With growing knowledge of gene interactions in the setting of disease, it is now well accepted that a complex disease phenotype is not a result of an aberration in a single effector gene; rather, it reflects various pathobiological processes that interact in complex networks [13]. Therefore, network-based approaches aid in the identification of disease genes, particularly in complex disease. Recently, a ‘network medicine’ approach has emerged as a powerful tool to systematically investigate the molecular complexity of disease etiology, which has ultimately led to the identification of disease-specific network clusters and pathways [14]. Thus, advances in these network-based approaches have uncovered the biological significance of disease-associated mutations and have aided in identifying new genes that can be targeted for therapeutic applications and/or serve as reliable biomarkers for disease progression [15,16].

Our present study proposes a network medicine approach to design an integrated gene and protein network and pathway-based analysis of IDD-associated genes. A comprehensive collection of genes shown to be associated with IDD was first performed from publicly available and well-informed databases. Functional enrichment analysis was then conducted based on identified IDD-related genes to ascertain their significant biological functions. An interaction network was also constructed to explore possible crosstalk among the significant genes related to IDD pathogenesis. We finally constructed a transcription factor (TFs)-targets regulatory network to gain more insight into the molecular mechanisms of disc degeneration. Our analysis identified RUNX1 as a potential key regulator of multiple signaling pathways involved in the etiology of IDD. These comprehensive and systematic analyses provide further valuable information for understanding the molecular mechanisms involved in IDD pathogenesis.

## 2. Materials and Methods

### 2.1. Collection of IDD-Related Genes

We collected all the genes which were reported in human genetic association studies deposited in PubMed. Key words used for association studies were Intervertebral disc degeneration/disease/displacement, lumbar disc degeneration/herniation. IDD-related genes were selected using the following criteria: (1) genes that were significantly associated with IDD (*p* < 0.05); (2) studies on human samples only; (3) genes with official symbols only, as un-annotated genes were excluded; (4) association studies must be reported in publications with reported association studies included. We also searched the GWAS (genome wide association studies) catalogue and open target databases to retrieve IDD-related genes [17]. As of September 1, 2019, a total of 284 genes with a significant association with IDD were retrieved and included in our present study.

### 2.2. Gene Ontology (GO) and Pathway Enrichment Analyses

Gene Ontology is a database that is used for the amalgamation of large-scale biological data that encompasses an organized, distinct and controlled vocabulary of gene annotation. The Kyoto Encyclopedia of Genes and Genomes (KEGG) database is a collection of online databases of genomes, enzymatic pathways and biological chemicals. The IDD-associated genes identified using the above search criteria were analyzed for functional enrichment of GO terms and pathways using STRING (Search Tool for the Retrieval of Interacting Genes), an online network cluster tool [18] as described previously [19,20]. Functional enrichment was performed in 3 categories of GO terms: Biological process (BP), molecular function (MF) and cellular component (CC). Kyoto Encyclopedia of Genes and Genomes (KEGG) pathway enrichment was also performed. All genes in the genome were used as the enrichment background. GO terms with a *p*-value of < 0.001, a minimum count of 5 and an enrichment factor of > 3.5 (the enrichment factor is calculated as ratio of the number of hit genes within a particular category to the total number of genes reported in same category) were collected. The significance of enriched pathways and *p*-values were calculated based on the cumulative hypergeometric t-test. False Discovery Rate (FDR) was used for multiple correction testing.

The enrichment of the top 15 GO terms based on FDR-corrected *p*-value was represented by ‘extended bubble plot analysis’, whereas KEGG pathways and associated genes were shown by Circos plot analysis. Circos plot is used for visualizing the data and information in a circular layout, which is used to demonstrate the relationships between genomic intervals or variation in genome structure. Circos plot was generated from command line using the table viewer plugin. Following integration of the KEGG pathways and associated gene sets, results data were converted into a contingency table for use with Circos. To simplify the interconnectivity between pathways and gene sets in the Circos plot, we performed filtering of data sets based on (1) limiting the KEGG pathways that are enriched for more than 10 genes (*n* > 10) and (2) limiting the gene sets whose enrichment was highly significant (FDR *p*-value ≤ 10^−9^, the genome wide significance). Therefore, KEGG pathways that represent less than 10 genes were not plotted and genes that are enriched in only one pathway were discarded to afford the plot simplification. Based on this filtering, we identified 24 genes and associated 12 KEGG pathways, and overlap between these enriched pathways and hallmark genes was represented by Circos plot.

### 2.3. Integrated Protein Network Analysis

To reveal the biological significance of enriched pathways and associated genes, we determined the functional interactions among proteins using integrated network analysis. The interaction between selected gene-encoded proteins was analyzed using the STRING (version: 11.0) database, a global resource to predict functional associations between proteins and cloud cluster networks [18]. All the genes shown to be associated with IDD were used as our input gene set and, again, the species was set as human. The Protein–Protein Interactions (PPIs) of enriched genes were analyzed for experimentally validated interactions, with a combined score of > 0.7 indicating a high confidence score for significant interaction. The nodes lacking a connection in the network were excluded. The interaction network was visualized by the Cytoscape (version: 3.7.1), a bioinformatics package for biological network visualization and data integration [21,22] as described previously [20]. The CytoNCA plug-in (version 2.1.6), was used to analyze the topological properties of nodes in the PPI network, and the parameter was set without weight [23]. In the interaction network, ‘circles/nodes’ indicate genes and ‘edges’ indicate the interaction between genes, and the number of edges that are connected to a node is the degree. To identify the most significant nodes, which are hereafter referred to as hub proteins, the connectivity degree of networks was assessed, and significance was calculated using the score ranking of each node. The proteins with a degree centrality of >5.0 were identified as hub proteins.

### 2.4. Network Module Analysis

To identify the most important clustering modules in the PPI network, we performed module/cluster analysis using the Molecular Complex Detection Algorithm (MCODE) plugin (version: 1.5.1), in Cytoscape [24]. Significant modules were identified according to the clustering score using the following criteria: ‘Degree cutoff = 2′, ‘node score cutoff = 0.2′, ‘Haircut = true’, ‘Fluff = false’, ‘k-core = 2′ and ‘max depth = 100′. The higher the clustering scores of the node, the more important the biological function of cluster is considered in disease pathogenesis. The threshold score of > 6 was chosen, therefore the first three clusters (Cluster 1, Cluster 2 and Cluster 3), with a clustering score of 12.9, 10.3 and 6.6, respectively, were selected and visualized with Cytoscape (version: 3.7.1). The interaction network for these clusters was made by mapping the ‘degree parameter’ to node size and color. Therefore, node size indicates the connectivity degree; larger circles indicate a higher degree. As the circles get bigger and their color changes from light to dark, the value of the connectivity degree of node gene increases. Therefore, node size and color changes indicate the value of the node gene/hub gene based on the parameter of connectivity degree. The higher degrees of hub genes in the interaction network signify the biological values of genes. The clustering modules with high node scores and connectivity degrees were considered as biologically significant clusters. The genes in the selected modules were subjected to Transcription factor (TF)-target gene regulatory network analysis to identify the potential genes relevant to IDD pathogenesis.

### 2.5. Prediction of Regulatory Networks of Transcription Factors (TFs)

To predict the coordinated regulation of the genes involved in cluster 1, we identified the candidate transcription factors that regulate these genes. To this end, we examined transcription factor binding motifs, which are enriched in the genomic regions of a query gene set and thus allow for the prediction of transcription factors using the iRegulon plugin (version 1.3), in Cytoscape [25]. To predict the transcriptional regulator and transcription factors using iRegulon analysis, we used genes from cluster 1 as input genes for motif and track search in the cis-regulatory control elements of genes. The criteria set for motif enrichment analysis were as follows: identity between orthologous genes ≥ 0.0, FDR on motif similarity ≤ 0.001, and TF motifs with normalized enrichment score (NES) > 3. The ranking option for Motif collection was set to 10 K (9713 PWMs) and a putative regulatory region of 20 kb centered around TSS (7 species) was selected for the analysis. Thereafter, TF-target pairs were obtained based on the TRANSFAC and JASPAR databases included in the iRegulon plugin. Our analysis yielded 126 significantly enriched motifs (normalized enrichment score NES > 3) that clustered into 47 groups by similarity, 32 of which were associated with transcription factors. In total, 32 transcription factors were predicted to potentially bind to these motifs.

### 2.6. Validation of Target Genes Using Publicly Available Microarray Datasets

To verify the predicted gene targets identified through MCODE analysis, we determined the mRNA expression profile of selected genes among degenerated and non-degenerated human discs. The gene expression profiles of 3 healthy and 3 degenerated discs were obtained from microarray datasets (GSE34095) available from the GEO database. The microarray data were conducted on the ‘Affymetrix Gene Chip Human Genome U133A Array’, which is comprised of more than 22,000 probe sets analyzing the expression level of 18,400 transcripts and variants, including 14,500 well-characterized human genes. The GEO datasets included RefSeq Gene ID, gene name, Gene symbol, microarray ID, adjusted *p*-value, and experimental group logFC relative to corresponding control groups. GEO2R was used to analyze the differential expression profiling between degenerated disc and non-degenerated controls. The FDR method was used to correct for multiple testing in GEO2R. Log fold change (FC) represented the fold change of gene expression, and *p* < 0.05 and logFC > 1 was set for statistically significant DEGs (differentially expressed genes). The heatmap for mRNA expression profiling of selected genes was generated by the R package of pheatmap. Additionally, unsupervised hierarchical clustering analysis of both experimental groups was performed using the Euclidean clustering distance and average clustering method. Further, to identify the overlap between degenerated and healthy human discs, we performed Principal Component Analysis (PCA) based on the expression profiles of selected genes using the ClustVis web tool [26].

## 3. Results

### 3.1. Identification of IDD-Related Genes

**Figure 1:**
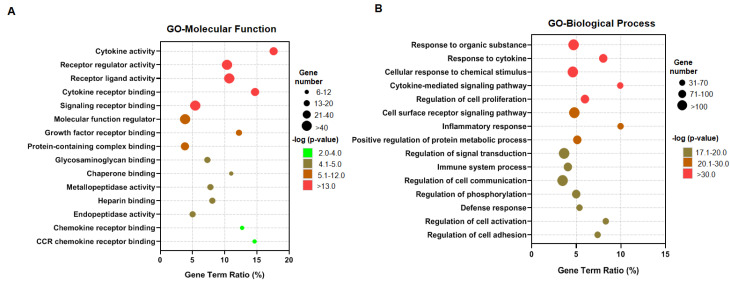
Functional annotation clustering by Gene Ontology (GO) and Kyoto Encyclopedia of Genes and Genomes (KEGG) pathway analysis identifies the enrichment of cytokine receptor signaling in Intervertebral disc (IVD) degeneration (IDD).GO analysis of IDD-related genes for (**A**) Molecular function and (**B**) Biological process. The top 15 enrichment pathways based on False Discovery Rate (FDR) *p*-value were shown in the bubble plot. The Y-axis label represents the pathway, and the X-axis label represents the gene term ratio (gene term ratio = gene numbers annotated in this pathway term/all gene numbers annotated in this pathway term). The size of the bubble represents the number of IDD-related genes enriched in the pathway, and color shows the corrected *p* value of the enriched pathway.

To perform integrated gene network analysis, we first screened genetic association studies related to IDD from the Pubmed database, GWAS catalogue and open target database. We selected only the publications that found gene(s) significantly associated with IDD (*p* < 0.05). We identified a gene set consisting of 284 genes that were significantly associated with IDD (Figure 1).

### 3.2. The Enrichment of Cytokine Receptor Signaling in IDD

To divulge the biological processes and pathways associated with genes related to IDD, we performed functional annotation clustering using GO and KEGG pathway analysis. The GO enrichment analysis showed that the IDD-related genes were involved in 73 GO terms for molecular function (MF) and 1587 terms for biological process (BP). The top 15 GO terms according to the ‘gene term ratio’ that designates the proportions of IDD-associated genes are shown in Supplementary Table S1. Our GO analysis for both molecular function and biological process showed that IDD-related genes were enriched in cytokine-mediated signaling pathways, cell surface receptor signaling pathways, inflammatory response pathways and chemokine receptor binding pathways (Figure 1A,B). These results suggest that cell signaling involved in cytokine and chemokine response pathways plays an important role in IDD pathogenesis.

**Figure 2:**
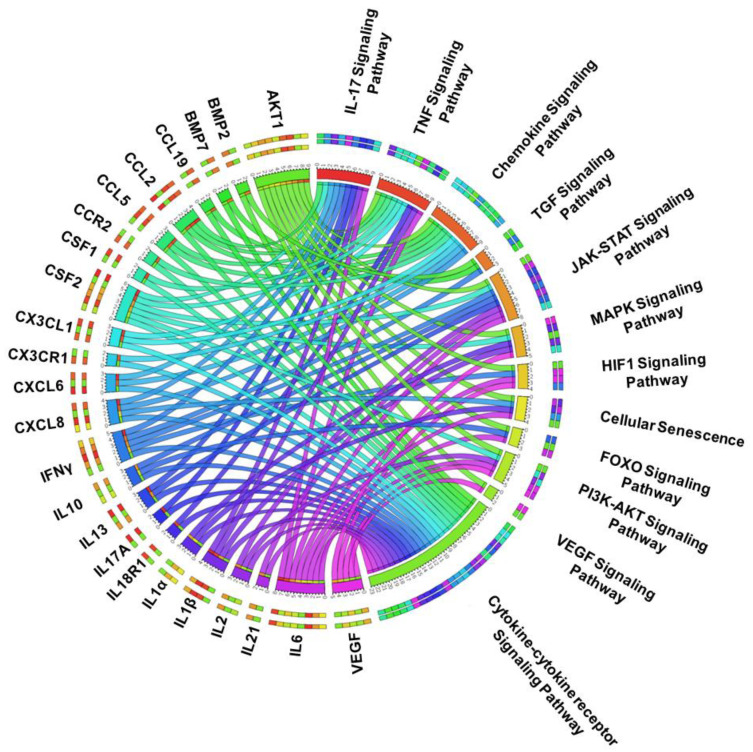
KEGG pathway enrichment analysis identifies the enrichment of inflammatory cytokine and IL17 signaling in IDD: The Circos plot represents significantly enriched pathways associated with regulated genes in the IDD detected using our functional enrichment analysis. The hallmark genes and associated pathways are color coded and were represented using a specific color in the inner ring. The ribbon/arc that originates from different genes and terminates at associated KEGG pathways demonstrates the connectivity of genes and KEGG pathways. However, the outer ring is composed of multiple color bars (color belonging to the inner ring represents specific genes and KEGG pathways) and represents different genes enriched in a specific pathway or different KEGG pathways shared by a single gene. The distinction between genes and pathways shown in circular layout were shown by the space between the two outer rings. When there is a space between the two outer rings, it demonstrates the representation of genes, whereas when both outer rings are connected, it represents the enriched KEGG pathway.

We next determined the potential function of these IDD-related genes using KEGG pathway enrichment analysis and our analysis identified the enrichment of a total of 111 KEGG pathways in 284 IDD-related genes. The interconnectivity between KEGG pathways and associated gene sets was shown by Circos plot analysis. To simplify the Circos plot, we performed filtering of data sets as described in the methods section and, therefore, KEGG pathways that represent less than 10 genes were not plotted, and genes that are enriched in only one pathway were also not included. Based on this filtering, 24 genes and 12 associated KEGG pathways were selected and overlap between these enriched pathways and hallmark genes was represented by Circos plot as shown in Figure 2. Our KEGG pathway enrichment analysis also found that “cytokine receptor interaction” is the most enriched pathway (Figure 2), which further supports the results of the GO analysis. Notably, the enrichment score analysis (gene term ratio) for the KEGG pathway showed that a large proportion (~15–20%) of IDD-related genes are involved in the IL-17 and TNF signaling pathways, suggesting that signaling genes involved in these pathways such as CCL2, IL-17A, CCL5, CSF1, CSF2, CX3CL1, IL18R1, IL1B, IL6, LIF, MMP14, MMP3, MMP9, PTGS2, SELE, SOCS3, TNF may play important roles in IDD development (Figure 2). Secondly, PI3K-Akt and Jak-STAT signaling pathways were significantly enriched, indicating that intracellular signaling in the regulation of cell cycle and cell proliferation are also important in disc degeneration (Figure 2).

### 3.3. PPI Network Analysis Reveals an Abundance of Organized Networks of Cytokine and Chemokine Signaling in IDD

Since genes work in synergy with other genes and involve complex interactions to perform biological functions, we evaluated the potential interactions among IDD-related gene-encoded proteins by mapping the genes using the STRING database. We restricted our PPI analysis to only experimentally validated interactions with a combined score of > 0.7 indicating highly significant interactions. Our analysis revealed that these genes formed a significant functional network that is involved in several essential biological processes, including chemokine and cytokine–receptor interaction pathways (Supplementary Figure S1). The PPI score in the interaction networks indicates the likelihood of functional linkage between two proteins. The topological properties of our resulting interaction network showed significant results during the validation phase, involving Topological Coefficients, Betweenness Centrality, Clustering Coefficient and Closeness Centrality (Supplementary Figure S2). Network analysis of the integrated gene interaction network revealed 0.483 Clustering Coefficient, 6 Connected Component, a Network Diameter of 9, a Network Radius of 1, 0.288 Network Centralization, 30120 (87%) Shortest Paths, 10.36 Average Neighbors, a Characteristic Path Length of 3.018, 0.056 Network Density and 1.192 Network Heterogeneity (Supplementary Figure S2).

**Figure 3:**
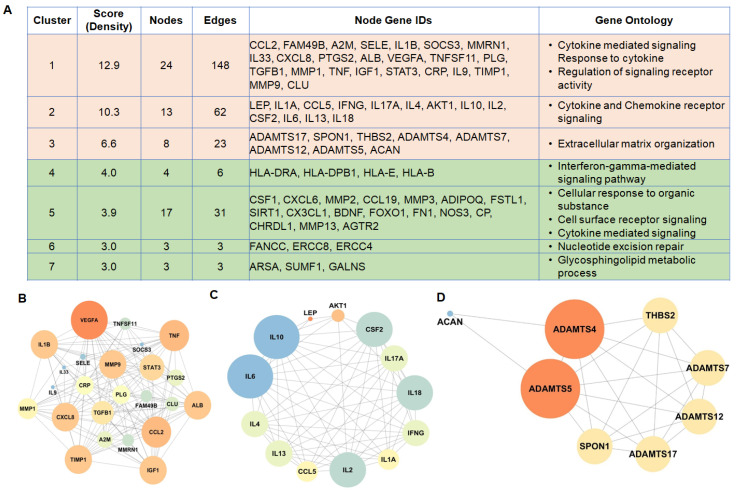
Cluster/module analysis in PPI network reveals the abundance of organized networks of cytokines and chemokines signaling: PPI network of IDD-related genes was constructed using the STRING database utilizing experimentally validated interactions with a combined PPI score of > 0.7. Sub-network analysis in the PPI network using MCODE identified the significant modules/clusters. (**A**) The cluster analysis in the PPI network resulted in 7 clusters, which include 72 nodes and 276 edges and enrichment of several GO terms for biological processes. (**B**) Cluster 1, (**C**) Cluster 2, and (**D**) Cluster 3 of the top three network clusters in the sub-network analysis of PPI networks of IDD-related genes. The cluster networks were visualized by Cytoscape (version: 3.7.1) by mapping the ‘degree parameter’ to node size and color. As the node size increased and color changed from light to dark, the value of the connectivity degree of node genes increased. All nodes are significantly enriched at an adjusted *p* value < 0.05.

To further prioritize the leading candidate genes involved in IDD pathogenesis, we performed a subnetwork analysis of the PPI network to identify significant modules/clusters. MCODE was used to perform cluster analysis in interaction networks, which represent the significant biological mechanisms leading to a specific phenotype/disease. The cluster analysis in the PPI network resulted in seven clusters that included 72 nodes and 276 edges (Figure 3A). Topological structure analysis revealed the score/density of nodes in the PPI network. Nodes with a higher score are more connected with other nodes, and they are designated as hub nodes, which contribute to the stability of the network. Our analysis revealed a total of three clusters with a score of >4. The interaction network was made by mapping the ‘degree parameter’ to node size and color. As node size in the interaction network got bigger and their color changed from light to dark, the value of the connectivity degree of the node gene increased. The higher degrees of hub genes in the interaction network indicated the biological significance of genes.

Cluster 1 exhibited the highest score, and 24 genes, such as CCL2, FAM49B, IL1B, SOCS3, CXCL8, ALB, VEGFA, TNF, IGF1, STAT3, TIMP1 and MMP9, were included (Figure 3B). Enrichment analysis demonstrated that the GO-BP terms enriched by these genes were associated with cytokine-mediated signaling pathways, pathways involved in cytokine response, as well as those associated with the regulation of signaling receptor activity (Figure 3A). Genes in cluster 2 primarily included IL10, IL6, IL18, IL2, IL17A and CCL5 (Figure 3C), which were enriched in GO-BP terms associated with cytokine and chemokine receptor signaling (Figure 3A). Genes in cluster 3 included ADAMTS4, ADAMTS17, ADAMTS5, SPON1, THBS2, ADAMTS7, ADAMTS12 and ACAN (Figure 3D), which were enriched in the extracellular matrix organization (Figure 3A). Taken together, our interaction analysis revealed the abundance of organized networks of cytokines and chemokines, indicating that perturbation of these signaling axes may be involved in IDD pathogenesis.

### 3.4. Transcription Factor–Target Gene Regulatory Network Analysis Predicts RUNX1 as a Major Driver of IDD

**Figure 4:**
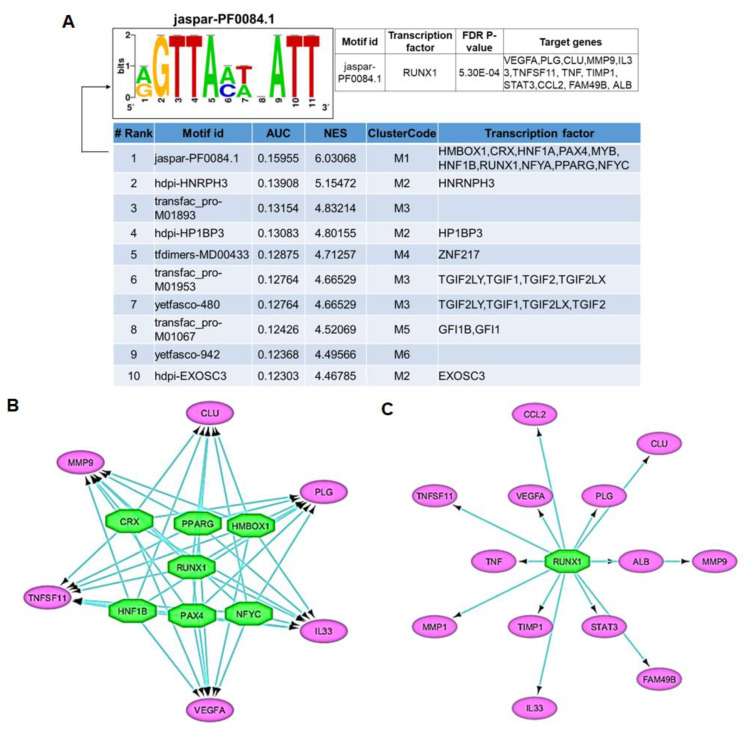
Transcription factor–target gene regulatory network analysis predicts RUNX1 as a major driver of IDD: Transcription factor (TF)-gene target regulatory network analysis was performed in network cluster 1 using the iRegulon plugin in Cytoscape (version: 3.7.1). (**A**) The iRegulon analysis showed the top transcription-binding motifs and their associated transcription factors found enriched in the cis-regulatory regions of the twenty genes of network cluster 1. (**B**) Regulatory network analysis represents the predicted transcriptional regulators/transcription factors (as octagonal nodes) of the target genes present in network cluster 1 (as oval nodes). To predict the transcriptional regulator using iRegulon analysis, the 24 genes from cluster 1 were used as input genes for motif and track search in the cis-regulatory control elements of genes. (**C**) The extended regulatory network analysis showed RUNX1 as the master regulatory transcription factor, which can potentially target a vast majority of the genes present in network cluster 1.

Because PPI-network analysis demonstrated that genes involved in cytokine and chemokine receptor signaling may mediate IDD pathogenesis, we next determined the coordinated regulation of these gene networks. To identify candidate transcription factors that regulate these genes, we performed transcription factor (TF)-gene target regulatory network analysis using iRegulon [25]. For this iRegulon analysis, we chose to use Cluster 1 because it was the most significant cluster from the above interaction network. All 24 genes (from cluster 1) were used as input genes for motif and track search in their cis-regulatory control elements to predict the transcriptional regulator and transcription factors. Using cis-regulatory sequence analysis of these 24 genes, we reverse-engineered the transcriptional regulatory network and predicted the candidate transcription factors that may control the regulation of these genes. Our analysis yielded 126 significantly enriched motifs (normalized enrichment score [NES] > 3) that clustered into 47 groups by similarity, 32 of which were associated with transcription factors and 15 were associated with tracks (Supplementary Figure S3A,B). In total, 32 transcription factors were predicted to potentially bind to the motifs present in genes involved in network cluster 1. To identify the best candidates from these transcription factors, several exclusion filters were applied including highest-ranking associated motif (> 3) and presence of number of targets (> 8), as well as maintenance of multiple motifs (> 4). These filters eliminated 19 of the 32 factors, leaving 13 transcription factors across 10 motif clusters and three tracks (Supplementary Figure S3A). Our analysis identified 10 transcription factors in rank 1 and one transcription factor each in rank 2 and 4 (Figure 4A). The transcription factor in rank 1/motif cluster 1 contains several novel transcription factors such as such as RUNX1, HMBOX1, CRX, HNF1B, PAX4, and NFYC that are involved in the transcriptional regulation of genes present in cluster 1 (Figure 4B). In addition, rank 1 also includes PPARG, which has been previously reported to regulate the inflammatory pathways in the nucleus pulposus of degenerated discs [27]. Our extended analysis suggests that among these candidate transcription factors, RUNX1 displayed the most significant network cluster (FDR-corrected *p*-value = 5.3E10^−4^) (Figure 4B). Moreover, the ‘regulator-target genes analysis’ demonstrated that RUNX1 potentially targets a vast majority of the IDD signature genes (54% = 13/24 gene) as identified in cluster 1 (Figure 4C). The predicted network identified specific interactions between regulators and target genes, including such targets of RUNX1 as FAM49B, VEGF, TNF, MMP9, TIMP1 and STAT3, etc. Together, our results suggest that the elements of this transcription factor–target gene regulatory network may be considered as a major gene driver, and thus likely contributing a significant role in IDD pathogenesis (Figure 4B,C).

### 3.5. Microarray-Based Expression Profiling Validates the Genes Identified in Network Clusters

**Figure 5:**
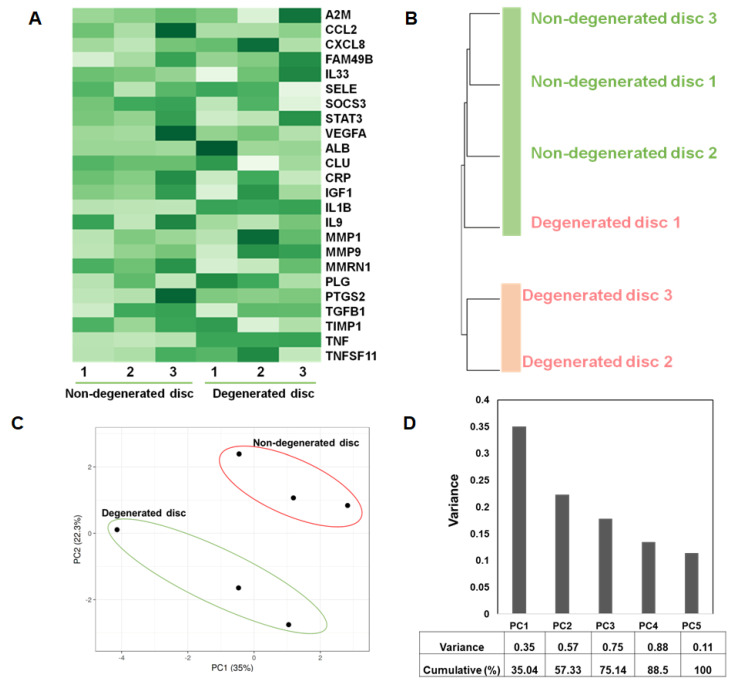
Microarray-based expression profiling validates the genes identified in network clusters: The expression of all twenty four genes identified in network cluster 1 was performed by analysis of microarray datasets (GSE34095) from three healthy and three degenerated human discs. Gene expression profiling was performed by GEO2R and the expression value for each gene was log2-transformed and then compared in a multivariate analysis via ClustVis using heatmap, hierarchical clustering and principal component analysis. (**A**) The heatmap visualization of mRNA expression of cluster 1 genes in healthy and degenerated human discs was performed using ‘ClustVis’. (**B**,**C**) The expression analysis of these twenty four genes led to the successful segregation of these cell populations by healthy vs degenerated via (**B**) hierarchical clustering and by (**C**) principal component analysis. (**D**) The PCA-plot shows PC1 and PC2 indicating 35% and 22.3% of the total variance, respectively.

Since cluster analysis of the interaction network indicates the relevance of cytokine and chemokine receptor signaling in IDD pathophysiology, we next determined the expression profile of these genes in independent datasets obtained from human discs that are also available in the public domain. To this end, all 24 genes were selected from the MCODE cluster 1 for their expression analysis in the microarray dataset (GSE34095), which included data from three healthy and three degenerated human discs. A heatmap analysis revealed differential expression of these genes, which discriminate well between healthy and diseased disc tissue (Figure 5A). The expression levels of CCL2, AKT1, STAT3 and VEGFA were significantly lower in the degenerated disc, whereas expression of CXCL8, IL2, FAM49B and PLG were significantly higher in degenerated human discs compared to healthy discs, suggesting that these genes might play a role in IDD pathogenesis (Figure 5A). We next evaluated whether changes in the expression profile of these 24 genes were sufficient to distinguish degenerated disc samples from non-degenerated control samples. For this, hierarchical clustering was performed and the resulting dendrogram clearly placed all three non-degenerated samples as one self-contained group distinct from two degenerated samples (disc 2, and 3) (Figure 5B). This result highlights that the expression profiles of 24 genes in network cluster 1 for non-degenerated discs are more similar to each other than the profile of degenerated samples indicating the possibility of IDD-specific gene expression. Although degenerated disc 1 clustered together with non-degenerated discs, this could be due to the variation in disease severity and degree of disc degeneration. Additionally, principal component analysis (PCA) was performed using these 24 differentially expressed genes. Interestingly, this analysis created two principal clusters that segregate the samples in accordance with their disease status: degenerated or healthy (Figure 5C). To further substantiate the correlation of variations of gene expression with IDD, we compared the resulting principal components with the disease status. The results showed that, together, PC #1 and PC #2 accounted for 57% of the variance in the data set and correlated well with IDD status of the subject (Figure 5D). Altogether, these results indicate that human intervertebral discs undergo reproducible and discernible transcriptomic changes of these 24 genes in the setting of IDD. Therefore, our analysis is indicative of distinct IDD-specific changes in the transcriptome of disc tissue, suggesting that these 24 genes (present in network cluster 1) may potentially constitute a transcriptomic signature of IDD pathogenesis.

## 4. Discussion

The present study utilized a network medicine approach through a comprehensive computational analysis to uncover the multiple molecular aspects of IDD. To date, the molecular mechanisms involved in the pathology of IDD are not fully elucidated. In our current study, we first identified 284 genes that were significantly associated with IDD from studies performed in human samples including nucleus pulposus, annulus fibrosus or whole disc. Our systematic analyses include functional annotation clustering using GO and KEGG enrichment analysis, establishment of interaction networks, network cluster/module analysis and TF-target gene network analysis to derive molecular insight that could help to reveal novel underlying mechanisms involved in IDD. The functional enrichment analysis indicated that genes were mainly involved in the GO terms of cytokine response, cell surface receptor signaling, inflammatory response and regulation of signal transduction, as well as in the KEGG pathways of cytokine–receptor interaction, IL17 signaling, PI3K-AKT and JAK-STAT signaling. The protein–protein interaction network and modules/cluster analysis again demonstrated that the inflammatory cytokine receptor and lL17 signaling may serve an important role in human IDD. In addition, regulatory network analysis identified multiple transcription factors including RUNX1, suggesting their role in the degenerative process of IDD. Altogether, the findings presented in this study indicate the involvement of inflammatory networks of chemokines and cytokines and several key transcription factors regulating these inflammasome in the pathogenesis of IDD, thus greatly enhancing our current understanding of the genetic mechanism of disc degeneration.

Our results of GO enrichment found that “response to cytokine and organic substance” and “inflammatory response and metabolic process,” both of which play important roles in inflammatory cascades, are related to IDD occurrence. Our results further show that inflammatory cytokine and related signaling may exist in the early development of IDD. Previous studies have indicated that degeneration is mediated by the abnormal production of pro-inflammatory molecules secreted by both nucleus pulposus and annulus fibrosus cells, as well macrophages, T cells and neutrophils [6,28,29,30,31]. The degenerative process of IDD is characterized by elevated levels of the inflammatory cytokines TNFα, IL-1 α/β, IL6 and IL17, which are known to be secreted by the disc cells themselves. Our analyses are in line with these published reports and further demonstrate the involvement of diverse etiological factors related to pro-inflammatory mediators in the pathogenesis of IDD, such as TNFα, IL1 α, IL6, IL17A, IL9, IL-33, IL4, IL10, IFNγ. These cytokines trigger a range of pathogenic responses by the disc cells that can promote autophagy, senescence and apoptosis, thus resulting in changes in cell phenotype and degeneration of matrices. The release of chemokines, such as CCL2, CCL5, CSF2, CXCL8, from degenerating discs promotes the infiltration and activation of T and B cells, as well as macrophages, which further amplify the inflammatory cascade and promote degeneration. A previous report by Gruber et al. demonstrated that disc cells produce IL17 in response to TNFα and IL1β stimulation and that its expression is increased in degenerate discs [32]. Furthermore, it was shown that IL17 acts synergistically with TNFα and IL1 in disc cells to enhance the inflammatory response [33]. Our results demonstrate the enrichment of IL17 signaling, further supporting the role of IL17 in the inflammatory cascade associated with IDD.

Degeneration is characterized by the complex interplay of several catabolic molecules involved in inflammatory processes leading to the loss of NP cellularity [34]. Here, we used a network-based approach to unravel the molecular basis of disc degeneration and prioritize the candidate genes that are likely responsible. Our PPI network analysis identified VEGF, TNF, TIMP1, IL1β, CCL2 and CXCL8 as hub genes with a high connectivity degree. Earlier studies showed elevated levels of several chemokines such as CCL3, CCL4, CCL5, MCP-3, MCP-4, and CXCL10 in degenerated and herniated discs [35,36]. The analysis of human discs indicated that CCL3 expression correlated positively with the grade of degeneration, and expression levels were higher in herniated tissue compared with healthy discs [36]. The cluster analysis indicated that the genes in cluster 1 and 2 were predominantly enriched in GO terms associated with cytokine-mediated signaling, response to cytokines, and inflammatory response. Our results indicated that 24 genes in cluster 1 with high degrees of interaction are candidate genes for disc degeneration, most of which have not been reported before. Our findings provide new perspectives for exploring the full picture of IDD candidate genes. For example, IL6, IL1β, and IL2, IL17A have been previously reported to be positively related to IDD, while we also found other genes that have not been previously implicated in IDD pathogenesis, including FAM49B, STAT3, and PLG. Importantly, we further demonstrated, with microarray analysis obtained from an independent dataset, that the expression of these genes was significantly changed in degenerated discs compared to healthy controls. These results point to the central role of inflammatory cytokines and chemotactic factors in the degenerative processes of the IVD.

To determine the coordinated regulation of identified candidate genes, we next identified TF–target gene regulatory networks using cis-regulatory element analysis. Our analysis identified seven transcription regulatory factors (RUNX1, CRX, PPARG, HMBOX1, HNF1B, PAX4 and NFYC) that likely control the regulation of the 24 genes involved in network cluster 1. Several features of this network are characteristic of master regulatory networks and may contribute to the development of IDD. The extended analysis of regulatory networks suggests the involvement of the RUNX1 transcription factor in the transcriptional regulation of a vast majority of the genes present in network cluster 1. Our analysis indicates that RUNX1 may be considered as a master regulator of IDD, as this transcription factor is highly integrated with other target genes and TFs through a plethora of interacting loops. Earlier studies demonstrate that RUNX1 is transiently expressed during condensations of mesenchymal cells [37,38], whereas RUNX2 and RUNX3 are robustly expressed in pre-hypertrophic chondrocytes [39]. An earlier study using a transgenic mouse model demonstrated the role of RUNX2 in the degeneration of IVD [39]. This study further showed that over-expression of RUNX2 or RUNX1 in cartilaginous endplates induces IVD destruction in mouse models. Our regulatory network analysis suggests that RUNX1 may contribute to the degeneration of discs, and thus may be involved in the development of IDD. Further studies using in vitro cell culture and valid in vivo animal models were needed to validate our findings and mechanistically support the role of RUNX1 in experimental models of disc degeneration.

## 5. Conclusions

In conclusion, the present study utilized a network medicine approach to provide insight into the pathogenesis of IDD and offers potential therapeutic targets for controlling the disease. The dysregulation of the cytokine/chemokine signaling axis and inflammatory response pathways are associated with IDD progression possibly through the activation of degrading proteases known to disrupt extracellular matrix organization. In addition, our regulatory network analysis suggests RUNX1 to be a master regulatory transcription factor that is involved in multiple pathological events in both the AF and NP. Since our study is based on information available from a public database, further experiments and clinical investigations are needed to confirm the results of the present study. Collectively, our study provides a systematic and comprehensive analysis to suggest the role of candidate genes, interacting pathways and transcription factors that may be involved in pathogenic events of disc degeneration. These potential candidate genes will improve our understanding of the genetic mechanisms of IDD, and their signature may eventually allow for the early diagnosis and the treatment of IDD. Future studies will further validate our findings through independent and rigorous experiments.

## Supplementary Materials

The following are available online at https://www.mdpi.com/2073-4425/11/7/771/s1, Figure S1: Protein–protein interaction (PPI) network of IDD-related genes formed a significant functional network. PPI network using the STRING database was constructed using experimentally validated interactions with a combined PPI score of >0.7, which indicates highly significant interactions among hub proteins. Figure S2: Topological properties of PPI network of IDD-related genes. Network analysis of PPI network using several topological features including: (A) Betweenness Centrality, (B) Topological Coefficients, (C) Average Clustering Coefficient and (D) Closeness Centrality. Figure S3: Transcription factor (TF)–gene target regulatory network motif analysis. The iRegulon analysis showed the (A) top transcription factors and (B) enriched track ID in the cis-regulatory regions of the twenty genes of network cluster 1. Table S1: Top 15 biological functions of IDD-related genes. Associated genes or Gene term ratio (%) = number of IDD-related genes/total number in the GO term X 100%; GO: Gene Ontology.
