# Supplementary material for: Network Analysis Identifies Gene Regulatory Network Indicating the Role of RUNX1 in Human Intervertebral Disc Degeneration
Source: Genes (Basel). 2020 Jul 9;11(7):771. doi: 10.3390/genes11070771 (PMC7397129; doi:10.3390/genes11070771)
Supplement: Supplementary file 1 [file genes-11-00771-s001.zip › Supplementray Materials/Table S1.docx]

**Supplementary Table 1: Top 15 biological functions of IDD‐related genes**. Associated genes or Gene term ratio (%) = number of IDD‐related genes/total number in the GO term X 100%; GO: Gene Ontology

| **GO ID** | **GO Term** | **Ontology Source** | **P-Value (FDR)** | **Gene Numbers** | **Associated Genes (%)** |
| --- | --- | --- | --- | --- | --- |
| GO:0097191 | Extrinsic apoptotic signaling | Biological Process | 5.58E-12 | 17 | 18.28 |
| GO:0005125 | Cytokine activity | Molecular Function | 1.90E-25 | 38 | 17.59 |
| GO:0005126 | Cytokine receptor binding | Molecular Function | 7.33E-25 | 40 | 14.71 |
| GO:0042379 | Chemokine receptor binding | Molecular Function | 2.50E-04 | 8 | 12.70 |
| GO:0070851 | Growth factor receptor binding | Molecular Function | 1.65E-08 | 16 | 12.21 |
| GO:0051087 | Chaperone binding | Molecular Function | 2.08E-05 | 11 | 11.00 |
| GO:0048018 | Receptor ligand activity | Molecular Function | 7.13E-25 | 49 | 10.70 |
| GO:0030545 | Receptor regulator activity | Molecular Function | 7.13E-25 | 50 | 10.35 |
| GO:0006954 | Inflammatory response | Biological Process | 3.68E-23 | 48 | 9.96 |
| GO:0019221 | Cytokine-mediated signaling | Biological Process | 6.73E-32 | 65 | 9.92 |
| GO:0031012 | Extracellular matrix | Cellular Component | 2.02E-11 | 26 | 9.19 |
| GO:0050865 | Regulation of cell activation | Biological Process | 8.46E-18 | 42 | 8.30 |
| GO:0005901 | Caveola | Cellular Component | 1.11E-02 | 6 | 8.11 |
| GO:0008201 | Heparin binding | Molecular Function | 4.29E-05 | 13 | 8.07 |
| GO:0008237 | Metallopeptidase activity | Molecular Function | 2.63E-05 | 14 | 7.78 |
