# Supplementary figures and images for: Network Analysis Identifies Gene Regulatory Network Indicating the Role of RUNX1 in Human Intervertebral Disc Degeneration
Source: Genes (Basel). 2020 Jul 9;11(7):771. doi: 10.3390/genes11070771 (PMC7397129; doi:10.3390/genes11070771)

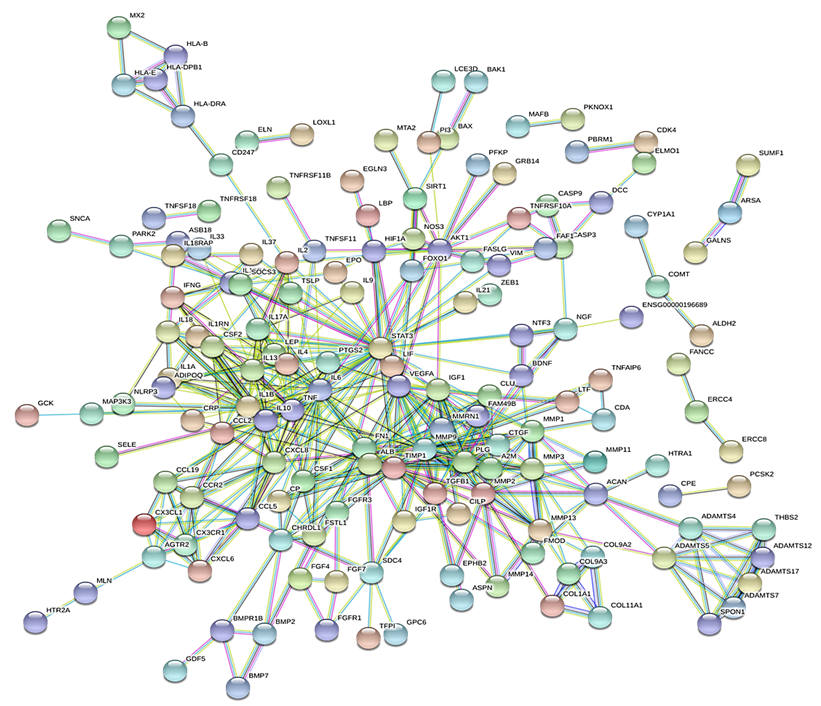

Supplement: Supplementary file 1 [file genes-11-00771-s001.zip › Supplementray Materials/Figure S1.tif]

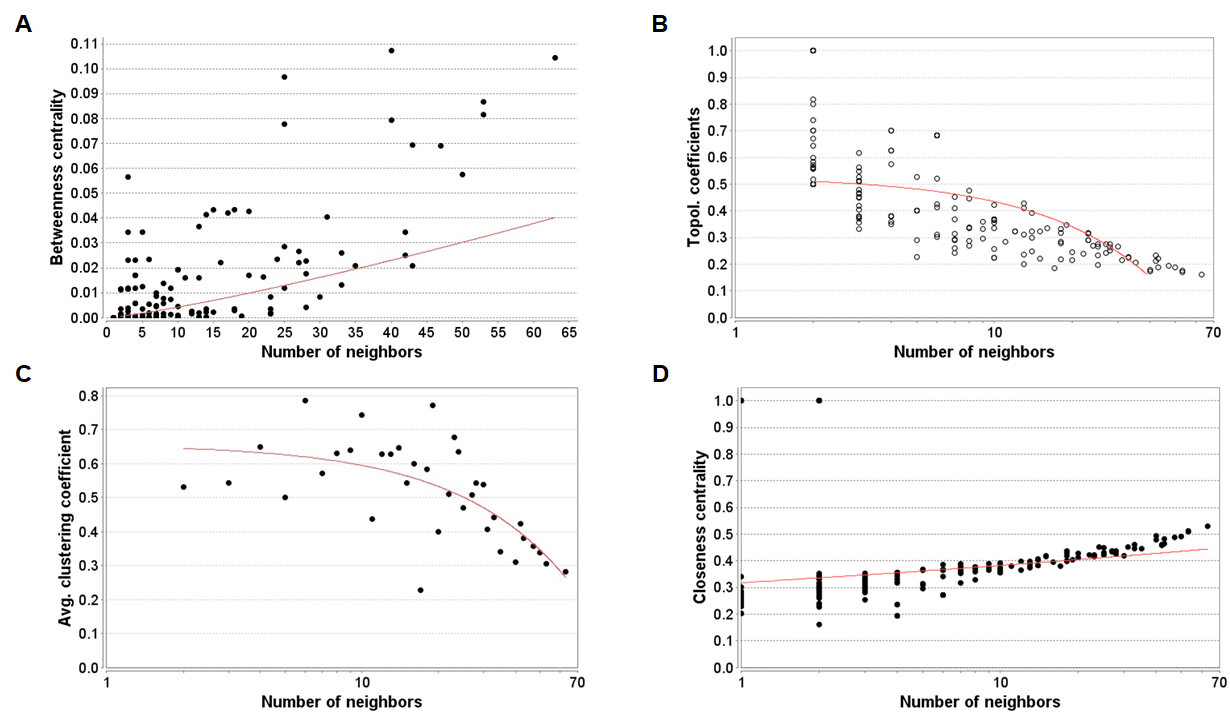

Supplement: Supplementary file 1 [file genes-11-00771-s001.zip › Supplementray Materials/Figure S2.tif]

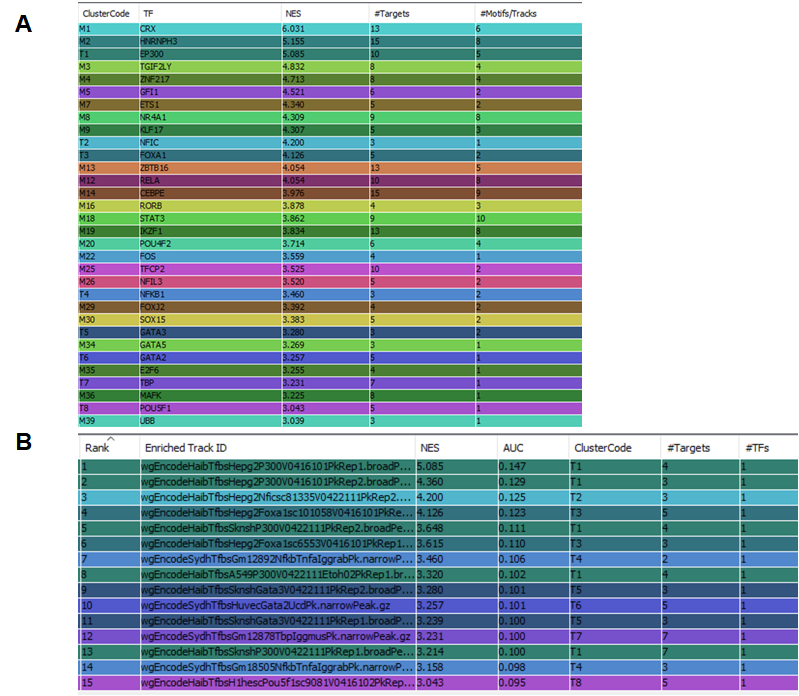

Supplement: Supplementary file 1 [file genes-11-00771-s001.zip › Supplementray Materials/Figure S3.tif]
